# The impact of age on goal-framing for health messages: The mediating effect of interest in health and emotion regulation

**DOI:** 10.1371/journal.pone.0238989

**Published:** 2020-09-17

**Authors:** Kouhei Masumoto, Mariko Shiozaki, Nozomi Taishi

**Affiliations:** 1 Graduate School of Human Development and Environment, Kobe University, Kobe, Hyogo, Japan; 2 Department of Applied Sociology, Kindai University, Higashiosaka, Osaka, Japan; 3 Faculty of Psychology, Doshisha University, Kyotanabe, Kyoto, Japan; 4 Japan Society for the Promotion of Science, Japan; University of Zurich, SWITZERLAND

## Abstract

Messages to promote health behavior are essential when considering health promotion, disease prevention, and healthy life expectancy. The present study aimed to examine whether (1) positive and negative goal-framing messages affect message memory and behavioral intention differently in younger, middle-aged, and older adults, (2) framing effects are mediated by interest in health (health promotion and disease prevention) and emotion regulation (cognitive reappraisal and expressive suppression), and (3) mediation effects differ between positive and negative frames. Participants (*N* = 1248) aged 20 to 70 years were divided into positive and negative frame conditions. Framing demonstrated interactive effects on message memory; all age groups showed higher recognition accuracy in the positive than the negative frame. The accuracy of younger adults was higher than that of older adults in the negative frame, while older adults showed higher accuracy than younger adults in the positive frame. Additionally, recognition accuracy was higher in the positive frame, as participants had higher interest in health promotion and used cognitive reappraisal more frequently. Contrariwise, emotion regulation and interest in health promotion did not have significant effects on memory in negative frames. Moreover, regardless of the message valence, age did not influence behavioral intention directly but was mediated by interest in health and emotion regulation, while the older the participants were, the higher their interest in health, resulting in higher intention. For emotion regulation, intention increased with higher reappraisal scores and decreased with increasing suppression. Our results suggest that interest in health and emotion regulation should be considered when examining the relationship between age and goal-framing for health messages.

## Introduction

Life expectancy has been increasing annually with the advancement of medical technology. As a result, the extension of healthy life expectancies has gained great importance in our lives, and health promotion and disease prevention have received increasing attention. Studies of suitable exercise, food, and lifestyle habits to promote health and prevent disease have been conducted in various fields such as public health, nutritional science, and health science. However, for older adults to engage in healthy behavior regardless of its type, the health message needs to interest them, remain with them, and motivate them.

### Goal-framing

Goal-framing has gained attention as a form of persuasive communication to increase the motivation for healthy or advocated behaviors. Goal-framing takes two forms: positive framing (PF), which emphasizes the benefit of engaging in a certain behavior, and negative framing (NF), which emphasizes the loss from not engaging in a certain behavior [1]. For example, in the context of healthy behavior, the PF would be, “if you exercise, you will gain a longer healthy life expectancy,” and the NF would be, “if you do not exercise, you will shorten your healthy life expectancy.” A study that examined the effects of framing in breast cancer screening showed that NF encouraged the participants to receive screening [2]. Apprehending how framed information affects judgment and behavior contributes not only to understanding framing effects but also the effective use of framing in health promotion campaigns [3]. However, results are inconsistent as to whether PF or NF is more persuasive because several factors are involved in goal-framing [4, 5].

### Age and frame messages

Previous studies have reported the effect of frame messages on individuals of different ages. For example, older adults had better recognition memory of PF than NF messages [6] and falsely remembered the NF messages as PF [6, 7]. Moreover, older adults who had received PF messages walked 17,000 more steps in a week than those who had received NF messages, and the effect of framing was sustained throughout the 28-day program [8]. These results regarding the enhancement of memory performance and health behavior in PF were explained using socio-emotional selectivity theory [9, 10], which posits that a future time perspective affects people’s goals and motivation. Older adults, when they consider their limited time horizons, are motivated by emotion regulation, increasingly value emotional meanings, and invest their cognitive and social resources in obtaining emotional value. As a result, the positivity effect of attending to and better memorizing positive information than negative information is observed [11, 12]. Therefore, older adults considered PF pamphlets more informative than NF pamphlets [13].

Meanwhile, another study showed that the emotions triggered by the PF message did not differ between younger and older adults, while the NF message triggered more negative feelings in younger adults [14]. Moreover, the results for physical affective response such as facial electromyography and skin-conductance level indicated a relationship between zygomaticus activity and subjective affective responses for PF in younger adults, but no relationship between physical response and subjective responses in older adults [14]. These results imply that younger adults are especially sensitive to NF messages; on the other hand, older adults are less affected by framing.

These studies indicate that the effect of framing is different in younger and older adults. However, few studies have touched on the impact of age difference on goal-framing, and its mechanism remains unclear [8]. Previous studies had insufficient sample sizes, and many of them compared younger and older adults, possibly overestimating the age difference in the goal-framing. Therefore, the primary purpose of the present study is to recruit a large sample size, including from the middle-aged population, and examine whether the effects of framed health-relevant messages on memory and behavioral intention differ between age groups.

### Mediation of the framing effect by interest and emotion regulation

**Fig 1:**
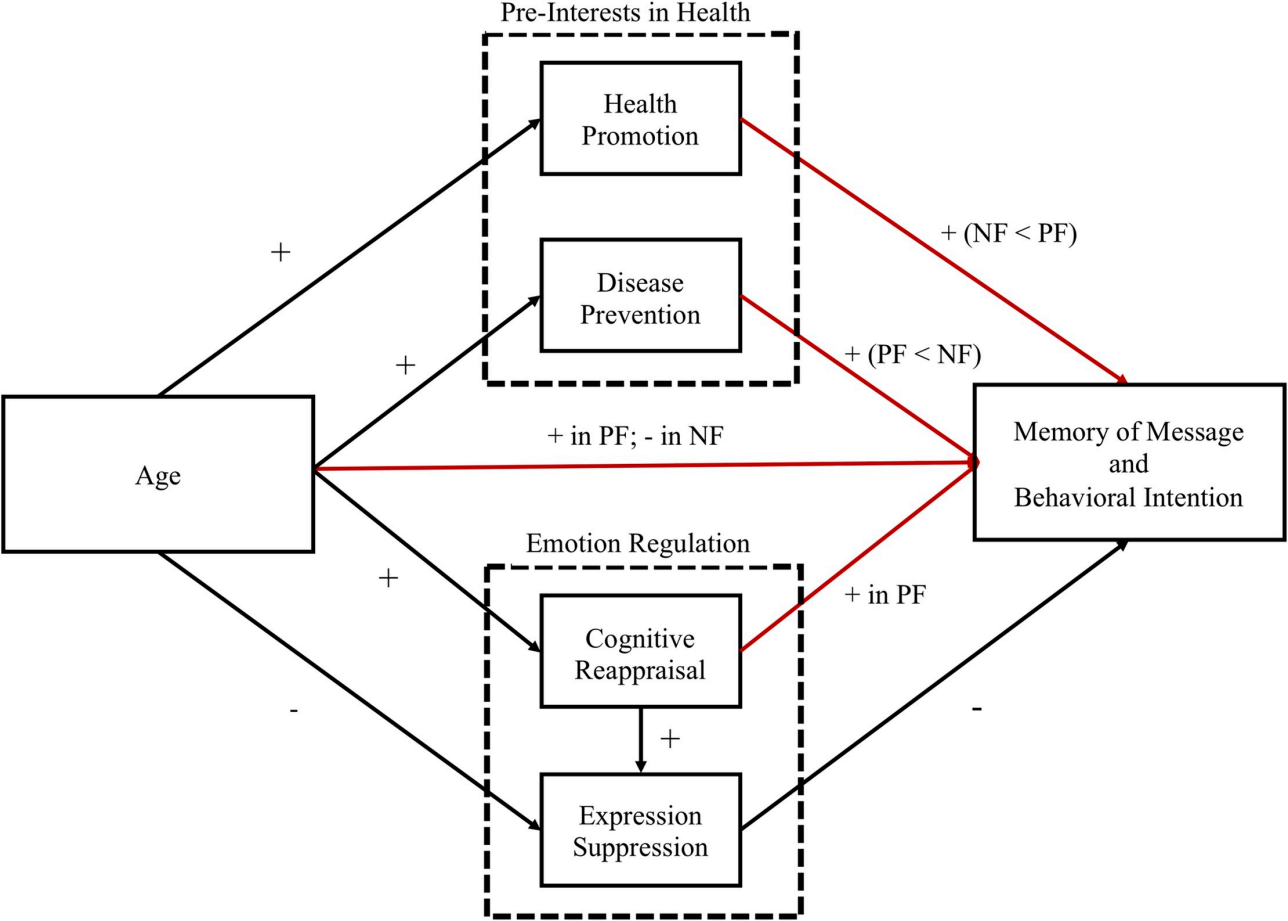
Hypothetical model in this study. PF represents positive frame, NF represents negative frame. + represents positive effect and − represents negative effect. Red means that the pass which expected to be different effect between PF and NF.

The second purpose of the present study is to examine whether the hypothetical model shown in Fig 1, in which the interests in health and emotion regulation serve as mediating parameters, differs between PF and NF.

#### Interest in health

Several meta-analyses of framing effects have yielded inconsistent results as to which type of framing is more effective [4, 5, 15]. Previous research suggested that personal involvement and interest in the content of health messages were factors influencing the framing effects; for example, NF is more persuasive when the self-involvement of the information is higher [16]. On the other hand, when self-involvement is low, the emphasis on benefit through PF is more persuasive. In particular, when the contents of messages are risk-associative and highly interesting to the individual, advocate judgment is performed in NF, while it is performed in PF when the interest is low [17]. In addition, as systematic processing takes place in cases of high interest, message recall and thoughts increase in NF [17].

In the present study, we measured two types of interest in health: interest in becoming healthier than at present (interest in health promotion) and interest in preventing diseases (interest in disease prevention). Older adults tend to have a higher interest in information on medical insurance, and younger adults generally have a lower interest in healthy daily habits [18]. Why do these interests in health change with age? The selection, optimization, and compensation model [19], known as a theory of life-span development, assumes three general functions of development: growth, maintenance, and regulation of loss. When young, the primary allocation of resource is directed toward growth. After adulthood, allocation shifts to maintenance. In old age, more resources are allocated to management or prevention of losses. In fact, a study of developmental changes in personal goal orientation [20] reported a shift in the direction of goals toward maintenance and loss prevention in the elderly. Thus, we expect that, although interest in health promotion and disease prevention will increase with age, interest in disease prevention will be more pronounced with age. As described above, advocate judgment is made under NF when interest is high, and therefore behavior intention and message memory should be enhanced under NF via interest in health.

#### Emotion regulation

Although the socio-emotional selectivity theory suggests that older adults are motivated by emotion regulation, few studies have also investigated the effects of age on framing effects using emotion regulation as a mediator.

The emotion regulation process model [21, 22] is widely accepted in the field of emotion regulation studies. According to this theory, emotion regulation includes an antecedent-focused strategy applied before the emotion occurs, and a response-focused strategy applied to the generated emotion reaction. Cognitive reappraisal (reappraisal) that changes the emotional impact by cognitively altering one’s interpretation of the emotion-eliciting situation is an antecedent-focused strategy. In other words, reappraisal is recognized as an adaptive strategy that reduces negative emotions and enhances positive emotions [22, 23]. Contrariwise, expressive suppression (suppression) that inhibits elicited emotions and expressive behavior is a typical response-focused strategy. This strategy is likely to lead a sense of self-inconsistency because of the differences between internally experienced emotions and externally expressed emotions [22]. Therefore, suppression is considered a maladaptive strategy that increases depression and anxiety [24, 25].

Several studies have reported that emotion regulation does not decline by age, but rather that reappraisal increases with age [26]. Positivity effects in the cognitive processing of older adults, such as memory and attention, are considered to be motivated by emotion regulation, which arises from the use of cognitive resources to achieve emotional satisfaction. Therefore, in this study, the path from age to reappraisal is expected to increase in strength with age, serving to facilitate memory and behavioral intention in PF. On the other hand, suppression requires self-monitoring and self-corrective action to inhibit the expression of emotion, and such processes demand cognitive resources [21]. We expected that the path from age to suppression will be negative as cognitive resources decline with age. In addition, the suppression will reduce behavior intention and message memory regardless of PF and NF because it is a strategy to inhibit emotional behavior.

## Materials and methods

### Ethics statement

We conducted an online panel survey through a marketing research agency (Macromill Inc. Tokyo, Japan) in order to avoid age and sex bias. The participants received an email containing the survey request. If they were willing to participate, they were asked to click on a link to the URL of the survey page. The first page of the survey clearly stated that the survey asks for personal information, that the responses are aggregated so as to maintain individuals’ anonymity, and that all the collected data would be used for research purposes only. If they agreed to participate, they provided informed consent by selecting a check box designated for the same. Informed consent was obtained from all participants. The Ethics Committee of the Graduate School of Human Development and Environment in Kobe University approved the study protocol (approval number 108).

### Participants

Participants were recruited from a large scale online panel database provided by Macromill Inc., which performs monthly veracity checks of respondents’ registration information and conducts a trap survey twice a year to identify and remove monitors providing groundless or contradictory responses. The participants were 1248 Japanese people aged 20 to 79 years (*M* = 49.29, *SD* = 16.76), and each age range (20s, 30s, 40s, 50s, 60s, 70s) had 208 people. Each age range was divided into two group such that for each valence, NF and PF, there were 104 people (men = 52, women = 52). Participants were assigned randomly to NF and PF. For their participation, the participants received points worth 100 Japanese Yen through the agency.

### Materials

#### Health-relevant messages

Although previous studies have utilized such messages for sexually transmitted disease, skin cancer, influenza, and cholesterol [13], sexually transmitted disease and skin cancer would not be common topics for most people in Japan. Therefore, we referred to public measures taken by the Japanese Ministry of Health, Labour, and Welfare for the general population and created four pamphlets about influenza, noroviruses, exercising, and diets. For each category, we prepared two types of pamphlets, NF and PF pamphlets. The difference between the NF and PF pamphlets was only in the framing of sentences, leaving other contents and sentences identical (see the Appendix 1).

Each pamphlet included four framing messages. The participants were presented one pamphlet of either valence in an order counterbalanced across participants. Participants in PF and NF were presented with all four pamphlets in a positive or negative frame, respectively. After reading each pamphlet, as a measure of behavioral intention, the participants rated how much they wanted to engage in each healthy behavior on a 7-point scale (1 = *not likely at all* and 7 = *very likely*).

For the memory task, we conducted a recognition task based on previous research [6, 13]. In the recognition task, the sentences in NF and PF with same message content were presented in pairs, and the participants judged whether the messages were presented in the PF or NF. There were four kinds of pamphlets with four framing messages in each pamphlet; therefore, a total of 16 recognition tasks were given to each participant. The paired sentence to recognize was randomly presented, and the arrangement of the PF and NF messages (i.e., which one was presented at the top) was counterbalanced.

#### Interest in health

To measure interest in health promotion, we presented the item, “I am interested in becoming healthier than I am right now,” while to measure interest in disease prevention, we presented the item, “I am interest in preventing diseases.” The participants scored their responses to each item on a 7-point scale (1 = *strongly disagree* and 7 = *strongly agree*).

#### Emotion regulation

We used the Japanese version [27] of the Emotion Regulation Questionnaire [22]. This questionnaire has 10 items (6 reappraisal items and 4 suppression items). The internal consistency, test-retest reliability, and construct validity of the Japanese version have been verified in a study involving undergraduate students. The participants rated each item on a 7-point scale (1 = *strongly disagree* and 7 = *strongly agree*).

### Procedure

First, we asked the participants to answer questions about their interests in health, then told the participants that we were studying effective advertisements for health promotion and disease prevention, for which they were to read four pamphlets. These pamphlets were presented on a web page for each topic. At the top of each pamphlet, we alerted the participants not to skip any part of the pamphlet by stating, “This is a survey about health. Although similar topics are presented repeatedly, please do not skip reading before you go on to the next question.” The order of the presentation of the pamphlets was counterbalanced between groups. Each time the participants finished reading one pamphlet, they answered the question on behavioral intention for the particular health behavior stated in the pamphlet.

After evaluating the pamphlets, they answered questions about emotion regulation, and then the recognition task was given. In the recognition task, the NF and PF sentences were presented in pairs, and the participants checked one of the two boxes before the sentence by clicking the option they thought they had seen in the pamphlets. They were not allowed to proceed to the next page without answering the items for all the selections on the webpage. Furthermore, in order to increase the reliability of the responses, if the participants chose the same answer for all items, a message appeared encouraging them to recheck their responses and correct them if necessary. It took about 15 minutes for the participants to answer all the questions.

### Data analysis

In order to examine the reliability of the average recognition and intention scores for the four pamphlets, we calculated Cronbach’s α, finding *α* = .93 for memory and *α* = .82 for intention, confirming adequate internal consistency for each variable. Therefore, we used the average scores for the four pamphlets as dependent variables.

For comparisons by age groups, we defined the participants in their 20s and 30s as the younger group (*n* = 416), the participants in their 40s and 50s as the middle-aged group (*n* = 416), and the participants in their 60s and 70s as the older group (*n* = 416). Table 1 shows the age, interests in health, and averages and standard deviations of emotion regulation in each age group for each valence. To confirm the homogeneity of age, emotion regulation, and interest in health between valence groups, we conducted a 2 × 3 (valence [NF, PF] × age group [younger, middle-aged, older]) analysis of variance (ANOVA) with age, interest in health, and emotion regulation as dependent variables. There were no significant main effects of age group or valence × age group interaction in any dependent variable (age: main effect of age group, *F* (2, 1242) = 5055.60, *p <* .001, *η*_g_^2^ = .90, main effect of valence, *F* (1, 1242) = .75, *p* = .39, *η*_g_^2^ = .00, interaction effect, *F* (2, 1242) = .10, *p =* .91, *η*_g_^2^ = .00; reappraisal: main effect of age group, *F* (2, 1242) = 1.14, *p* = .32, *η*_g_^2^ = .00, main effect of valence, *F* (1, 1242) = 2.76, *p* = .10, *η*_g_^2^ = .00, interaction effect, *F* (2, 1242) = 36.73, *p =* .91, *η*_g_^2^ = .00; suppression: main effect of age group, *F* (2, 1242) = 1.51, *p =* .22, *η*_g_^2^ = .00, main effect of valence, *F* (1, 1242) = .48, *p* = .49, *η*_g_^2^ = .00, interaction effect, *F* (2, 1242) = .97, *p =* .38, *η*_g_^2^ = .00; health promotion: main effect of age group, *F* (2, 1242) = 3.87, *p <* .05, *η*_g_^2^ = .01, main effect of valence, *F* (1, 1242) = .04, *p* = .84, *η*_g_^2^ = .00, interaction effect, *F* (2, 1242) = .83, *p =* .44, *η*_g_^2^ = .00; disease prevention: main effect of age group, *F* (2, 1242) = 50.66, *p <* .001, *η*_g_^2^ = .08, main effect of valence, *F* (1, 1242) = .01, *p* = .94, *η*_g_^2^ = .00, interaction effect, *F* (2, 1242) = 2.90, *p =* .06, *η*_g_^2^ = .00). These results indicate no significant differences in age, emotion regulation, or interest in health between the valence groups.

**Table 1:** Means and standard deviations of age, interest in health, and emotion regulation by age group and frame valence.

Variables		Younger		Middle-aged		Older	
		PF (*n* = 208)	NF (*n* = 208)	PF (*n* = 208)	NF (*n* = 208)	PF (*n* = 208)	NF (*n* = 208)
Age		29.98 (5.47)	30.12 (5.66)	48.79 (5.80)	49.26 (5.64)	68.68 (5.50)	68.88 (5.25)
Interest in Health	Health promotion	5.36 (1.70)	5.33 (1.73)	5.44 (1.51)	5.34 (1.57)	5.54 (1.38)	5.71 (1.46)
	Disease prevention	4.09 (1.42)	4.23 (1.50)	4.47 (1.38)	4.19 (1.50)	5.02 (1.34)	5.15 (1.35)
Emotion Regulation	Cognitive reappraisal	4.29 (.95)	4.15 (.91)	4.25 (.90)	4.26 (.77)	4.37 (.74)	4.25 (.74)
	Expressive suppression	3.93 (1.17)	3.90 (1.17)	3.92 (.95)	3.98 (1.07)	4.11 (.94)	3.97 (.94)

PF = positive frame; NF = negative frame.

Next, we conducted a 2 × 3 (valence [NF, PF] × age group [younger, middle-aged, older]) ANOVA to examine the effects of age and framing on message memory and intention for healthy behavior, then calculated the zero-order Pearson correlations of the measured variables and conducted structural equation modeling (SEM) analysis to examine whether the hypothetical model (Fig 1) fit. After confirming the validity of the model, we examined whether each path between the variables differed between PF and NF using multiple group analysis.

## Results

### Memory of health message

**Fig 2:**
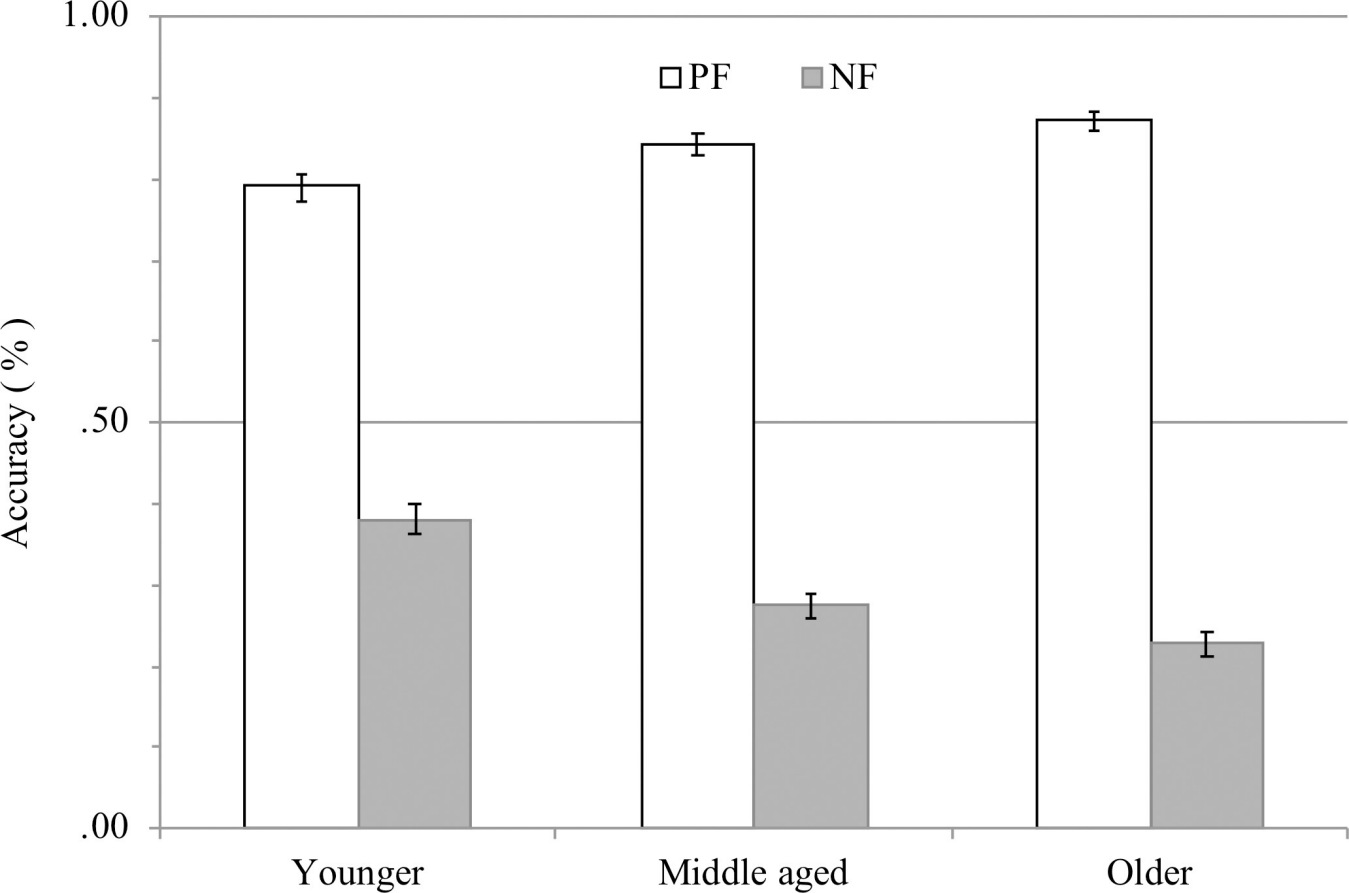
Memory of message (recognition performance) by age group. PF represents positive frame, NF represents negative frame. Error bars show standard error of the means.

A 2×3 ANOVA showed that the main effects of age group, *F* (2, 1242) = 3.51, *p* < .05, *η*_g_^2^ = .01, and valence, *F* (1, 1242) = 2245.16, *p* < .001, *η*_g_^2^ = .64, and their interaction effect, *F* (2, 1242) = 36.73, *p* < .001, *η*_g_^2^ = .06, were significant (see Fig 2). Since the interaction effect was significant, a simple main effect test was conducted, finding it to be significant for age group for both PF and NF, *F* (2, 1242) = 8.83, *p* < .001, *η*_g_^2^ = .01; *F* (2, 1242) = 31.41, *p* < .001, *η*_g_^2^ = .05, respectively. Multiple comparison revealed that, in PF, the score of the younger group was significantly lower than that of the middle-aged group, *p* < .05, and the older group, *p* < .001. On the other hand, in NF, the recognition scores were higher in the order younger, middle-aged, and older groups, *p* < .01. Moreover, for any age group, the simple main effect of valence was significant, and the recognition score of PF was significantly higher than that of NF: younger, *F* (1, 1242) = 429.72, *p* < .001, *η*_g_^2^ = .26; middle-aged, *F* (1, 1242) = 824.92, *p* < .001, *η*_g_^2^ = .40; older, *F* (1, 1242) = 1063.98, *p* < .001, *η*_g_^2^ = .46.

Since the recognition task in the present study required choosing from two options, the average scores on recognition accuracy were compared with chance level (50%). A series of one-sample *t*-tests revealed that for all age groups, the recognition score was significantly higher than chance level in PF (younger, *t* (207) = 22.02, *p* < .001, *r* = .84; middle-aged, *t* (207) = 29.76, *p* < .001, *r* = .90; older, *t* (207) = 37.08, *p* < .001, *r* = .93) and significantly lower than chance level for NF (younger, *t* (207) = −6.56, *p* < .001, *r* = .42; middle-aged, *t* (207) = −14.69, *p* < .001, *r* = .71; older, *t* (207) = −19.20, *p* < .001, *r* = .80).

### Behavioral intention

**Fig 3:**
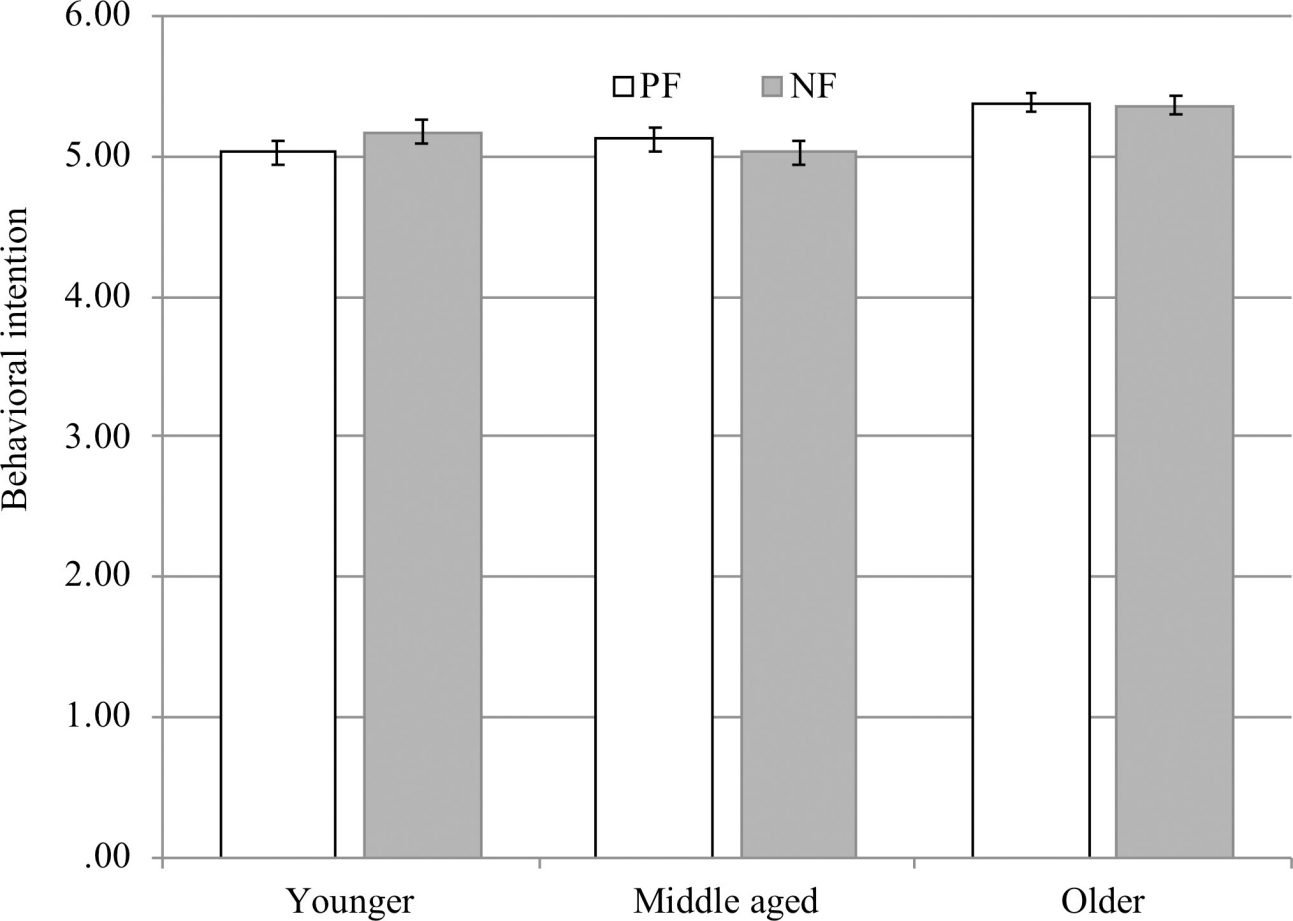
Intention for healthy behavior by age group. PF represents positive frame, NF represents negative frame. Error bars show standard error of the means.

A 2×3 ANOVA showed the main effect of age group to be significant, *F* (2, 1242) = 8.07, *p* < .001, *η*_g_^2^ = .01; however, the main effect of valence, *F* (1, 1242) = .02, *p =* .88, *η*_g_^2^ = .001, and the interaction effect of age group × valence, *F* (2, 1242) = 1.10, *p* = .33, *η*_g_^2^ = .002, were not significant (see Fig 3). Post hoc analysis of the main effect of age revealed that the older group scored significantly higher in intention than the younger group, *p* < .001, and the middle-aged group, *p* < .01. This result implies that, regardless of NF and PF, older adults have higher intention for healthy behavior.

### Analysis of the model with intention and emotion regulation as mediating variables

Table 2 shows the zero-order Pearson correlations of the measured variables. We conducted SEM with the hypothetical model shown in Fig 1, using memory of message and behavioral intention as dependent variables.

**Table 2:** Zero-order Pearson correlations of the measured variables by the valence.

Variables			Mean	SD	B	C	D	E	F	G
A. Age		Total	49.29	16.76	.07**	.28**	.05	.03	.10**	−.04
		*PF*	*49*.*15*	*16*.*77*	.*06*	.30**	.*05*	.*06*	.14**	.22**
		**NF**	**49.42**	**16.77**	.09*	.25**	**.05**	**.00**	**.06**	−.26**
Interest in Health	B. Health Promotion	Total	5.45	1.57	1	.43**	.07*	.02	.49**	.04
		*PF*	*5*.*44*	*1*.*54*	*1*	.43**	.09*	.*02*	.51**	.17**
		**NF**	**5.46**	**1.6**	**1**	.43**	**.05**	**.02**	.48**	**.00**
	C. Disease Prevention	Total	4.53	1.47		1	.21**	.06*	.39**	.03
		*PF*	*4*.*53*	*1*.*43*		*1*	.29**	.*08*	.42**	.15**
		**NF**	**4.52**	**1.51**		**1**	.12**	**.04**	.37**	**−.03**
Emotion Regulation	D. Reappraisal	Total	4.26	0.84			1	.42**	.19**	.05
		*PF*	*4*.*3*	*0*.*87*			*1*	.41**	.22**	.14**
		**NF**	**4.22**	**0.81**			**1**	.42**	.16**	**−.07**
	E. Suppression	Total	3.97	1.05				1	−.01	−.01
		*PF*	*3*.*99*	*1*.*03*				*1*	.*00*	.*00*
		**NF**	**3.95**	**1.06**				**1**	**−.01**	**−.07**
F. Intention for Healthy Behavior		Total	5.18	1.16					1	.04
		*PF*	*5*.*18*	*1*.*14*					*1*	.19**
		**NF**	**5.19**	**1.18**					**1**	**.00**
G. Recognition of Health Message		Total	0.57	0.34						1
		*PF*	*0*.*84*	*0*.*17*						*1*
		**NF**	**0.29**	**0.24**						**1**

PF = positive frame; NF = negative frame

*p < .05

**p < .01.

#### Memory of health message

**Fig 4:**
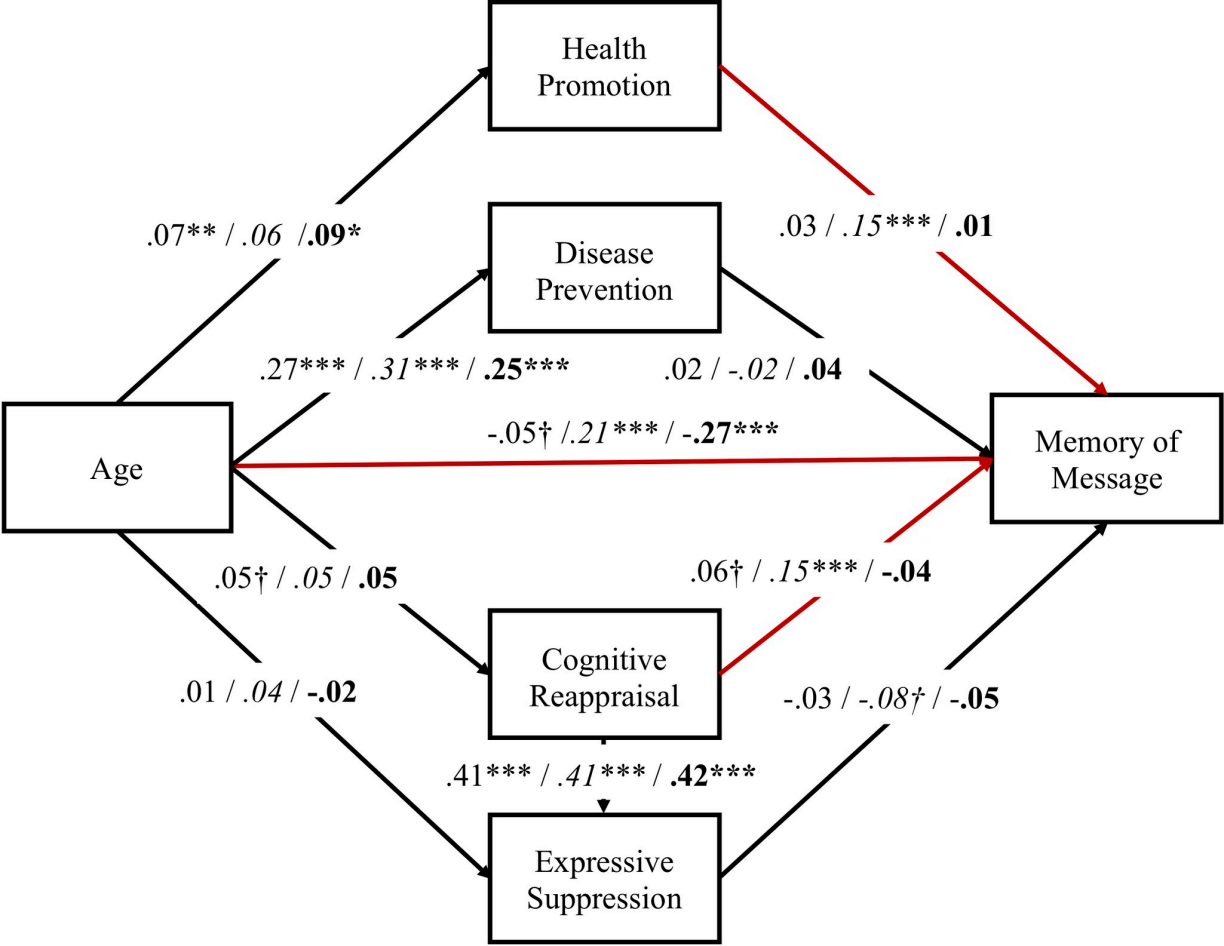
Estimates from structural equation modeling of memory of health message, and multi-group analyses of the differences between positive and negative frames. Standardized path coefficients are presented for total before the first slash, for positive frame (in italic) before the second slash, and for negative frame (in bold) after the second slash. Red lines showed the significant difference between positive and negative frame. ^†^*p <* .10. * *p* < .05. ** *p* < .01. ***p < .001.

Results of SEM indicated sufficient goodness of fit with memory score as an outcome (Fig 4), χ^2^ (3) = 7.04, *p* = .07, GFI = .998, CFI = .991, RMSEA = .033, AIC = 43.04. Multiple group analysis (Fig 4) revealed that the effects of age on interest and emotion regulation did not differ between PF and NF, and the older the participants, the higher their interest in health, particularly health promotion. Moreover, the path from age to reappraisal was marginally significant (*p* < .10), and no age effect was observed on suppression.

The direct path from age to memory reversed between the frames (*p* < .01); in PF, the memory score was higher in older participants; contrariwise, the memory score was higher in younger participants in NF. Moreover, the paths from interest in health promotion to memory (*p* < .05) and from reappraisal to memory (*p* < .05) showed significant differences between frames. In PF, memory scores were higher as people had a higher interest in health promotion and higher rates of using a reappraisal strategy. On the other hand, in NF, reappraisal and the interest in health promotion did not have significant effects on memory. These results indicate that the effects of framing on memory of health-related messages in PF are mediated by interest in health promotion and reappraisal strategies to promote memory. Contrariwise, in NF, neither interest nor emotion regulation affects memory as a mediator.

#### Behavioral intention

**Fig 5:**
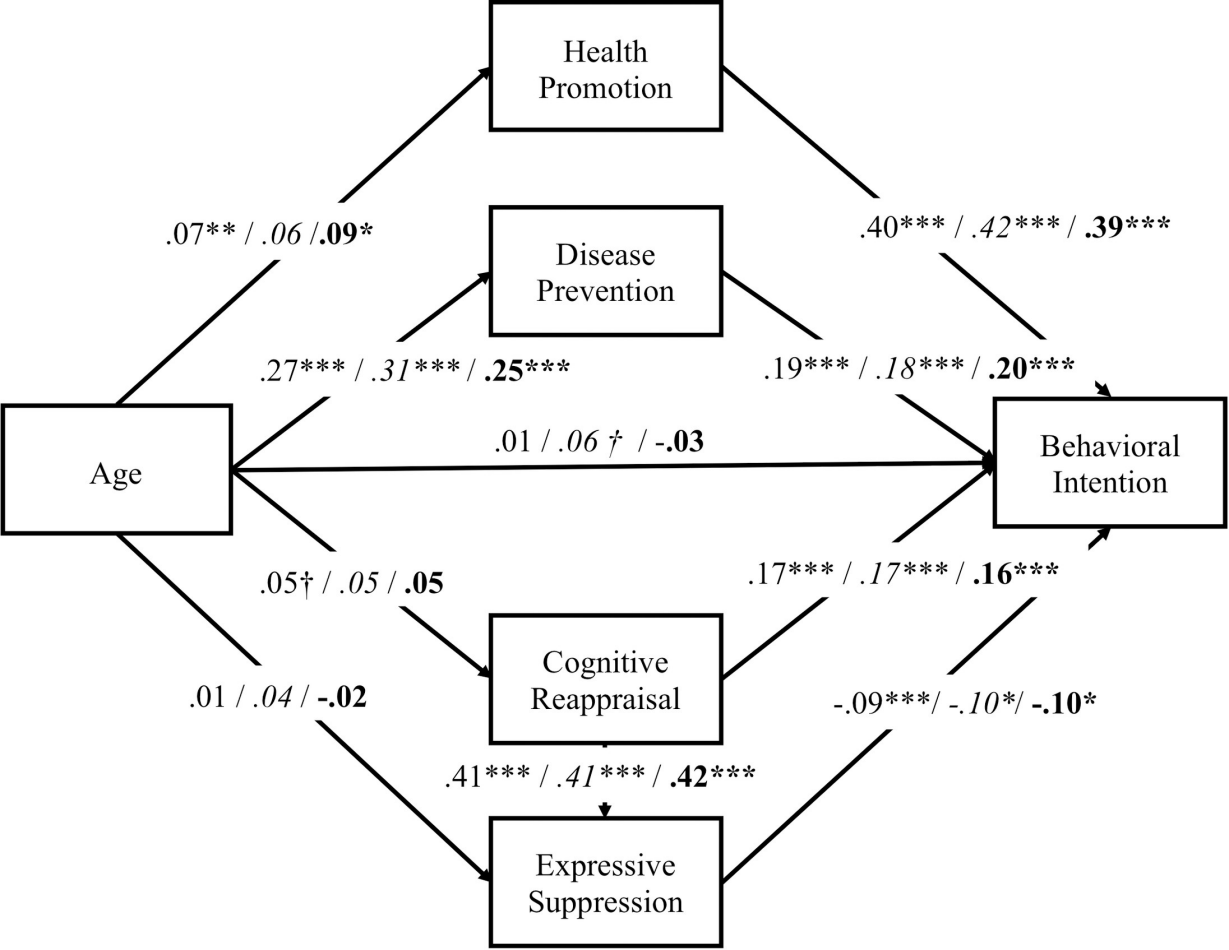
Estimates from structural equation modeling of behavioral intention, and multi-group analyses of the differences between positive and negative frames. Standardized path coefficients are presented for total before the first slash, for positive frame (in italic) before the second slash, and for negative frame (in bold) after the second slash. ^†^*p <* .10. * *p* < .05. ** *p* < .01. ***p < .001.

We conducted SEM with intention as an outcome (see Fig 5), and sufficient goodness of fit was attained, *χ*^*2*^ (3) = 7.04, *p* = .07, GFI = .998, CFI = .993, RMSEA = .033, AIC = 43.04. Multiple-group analysis found no significant differences between valences in any path. Regardless of message valence, age did not directly influence intention, but indirectly affected intention via mediation by interest in health and emotion regulation. For interest in health, the older the participants were, the higher their interests in health, resulting in higher intention. For emotion regulation, intention increased as the reappraisal score increased and decreased as suppression increased.

## Discussion

The purposes of the present study were to determine (1) the effect of age on goal framing, (2) whether the effects of age on message memory and behavioral intention are mediated by interest in health and emotion regulation, and (3) whether the effects of mediation differ between NF and PF.

### Memory of health message

The results of ANOVA revealed no main effect of age group on recognition score in general without division into PF or NF. This result was consistent with previous studies that reported that memory declines with age are prominent in recall but not recognition [28].

ANOVA also showed a significant interaction between age group and valence. With regard to the age difference for each valence, as per our hypothesis, the scores in NF and PF were higher in the younger group and older group, respectively. Previous studies examining emotional memory have found a negativity bias, whereby younger adults remember negative information more than positive information [29], and a positivity effect, whereby older adults remember positive information more than negative information [12]. Additionally, the positivity effect occurs because older adults are motivated by emotion regulation, investing cognitive resources to achieve emotional goals [9]. Thus, we predicted that the more adaptive emotion regulation (reappraisal) was used with age, the better the memory of the positive message in PF would be. Multiple group analysis (Fig 4) showed that reappraisal enhanced memory performance in PF, although the effect of age on reappraisal was small. On the other hand, suppression was predicted to decrease with age: Inhibiting the expression of emotion requires self-monitoring, which demands cognitive resources [21], and the path from age to suppression was not significant. Some cross-cultural studies have revealed cultural differences in suppression [30, 31]. In East Asian countries such as Japan, people value interpersonal relationships and place high importance on self-control of thoughts and behaviors, and suppression is necessary to determine emotional responses that best fit a social context [31]. We consider that an effect of age on suppression was not observed because, as the participants in this study, all Japanese, use suppression on a daily basis, cognitive resources are not required to use suppression.

Regarding the goal-framing effect on each age group, recognition accuracy was better in PF than in NF regardless of age. The recognition task in the present study required the participants to judge whether the messages were presented in PF or NF. As the investigators did not tell the participants about the recognition task before presenting the messages, the recognition task in this study was particularly difficult for them. However, we note that they appear not to have performed the recognition task randomly because the recognition accuracy was above chance level in all age groups for PF and below chance level in all age groups for NF. Why then were there differences in recognition performance between PF and NF? This could be due to interest in health. Previous studies of memory accuracy and distortion reported that although people can remember the gist and general contents of information, the recollection of the details of the information depends on an individual’s schemas, goals, and motivations [32, 33], and self-involvement and interest promote cognitive processing such as memory [17]. The participants of the present study had a higher interest in health promotion (mean = 5.45, *SD* = 1.57) than in disease prevention (mean = 4.53, *SD* = 1.47). A high interest in health promotion would facilitate the judgement that a message presented in PF emphasized the benefit of engaging in a certain behavior for their health. Thus, the recognition score was higher than chance level in PF and lower than chance level in NF. Additionally, we predicted that the effects of interest in health on memory of messages would differ between PF and NF. The results of multi-group analysis also supported our hypothesis of interest in health promotion: The higher the health promotion, the better the recognition of messages in PF. Also, as expected, interest in disease prevention increased with age, which could be interpreted as indicating a shift in goal orientation to maintenance/loss prevention with age [20]. However, interest in disease prevention did not affect recognition in either PF or NF. As mentioned above, since the interest in health promotion was higher than that in disease prevention, it would appear that interest in health promotion negated the mediation effect of the interest in disease prevention.

On the other hand, neither emotion regulation nor interest in health mediated the effect of age on memory performance in NF. This result is possibly because the older adults did not engage in negative information at the point of information input. Isaacowitz and Choi [34] compared the attention toward health information between younger and older adults and showed that unlike younger adults, older adults did not pay attention to negative content and controlled their feeling earlier. They indicated the possibility that older adults apply an efficient looking strategy that extracts important information without engaging visually with negatively valenced materials [34].

### Intention for healthy behavior

There was no framing effect on behavioral intention. As hypothesized, the results of SEM (Fig 5) showed that people with a higher interest in health and more frequent use of reappraisal showed increased behavioral intention, and that suppression inhibited behavioral intention. However, multigroup analysis showed no difference between PF and NF in any paths.

The difference in the results for behavioral intention and memory could be due to there being little framing effect on behavioral intention. Meta-analyses that distinguished the goal framing outcome into attitudes, intentions, and behavior found no effect of framing when persuasion was assessed by attitudes/intentions, and there was a dissociation between changes in attitudes/intentions and changes in actual behavior [4].

## Conclusion and limitations

There have been no consistent results in goal-framing studies regarding which framing, PF or NF, is more persuasive. A meta-analysis of the memory of framing message revealed that, though the effect size was small, memory performance was better in PF than NF [5]. Our results showed that the age differences in the memory of health-related messages vary between PF and NF, suggesting that we need to consider interest and emotion regulation when examining the relationship between age and goal-framing effects.

On the other hand, this study examined the effect of age cross-sectionally, and a longitudinal study is necessary to strictly examine the effect of aging. Additionally, the present study did not examine whether the behavior actually changed depending on the framing message, and we used measures like self-ratings that might show age differences in metacognitive ability, introspective abilities, and response tendencies. Therefore, a large-scale study is required in the future that includes behavioral indices. Moreover, we could not judge whether the participants used reappraisal or suppression at the time of message presentation because the present study examined emotion regulation using a questionnaire. Experimental studies manipulating emotion regulation are necessary to examine how emotion regulation affects goal-framing.

## Supporting information

S1 Appendix (DOCX)
