# Supplementary material for: The impact of age on goal-framing for health messages: The mediating effect of interest in health and emotion regulation
Source: PLoS One. 2020 Sep 17;15(9):e0238989. doi: 10.1371/journal.pone.0238989 (PMC7498008; doi:10.1371/journal.pone.0238989)
Supplement: S1 Appendix — (DOCX) [file pone.0238989.s001.docx]

**S1 Appendix**

**The positive and negative-frame messages in the norovirus pamphlet (Translated from Japanese)**

**What is the disease caused by norovirus?**

(Positive Frame 1) *With proper knowledge of noroviruses, it is possible to prevent food poisoning and infectious diseases caused by norovirus.*

(Negative Frame 1) *Without proper knowledge of noroviruses, it is not possible to prevent the occurrence of food poisoning and infectious diseases caused by norovirus.*

Norovirus can be transmitted to the mouth by the fingers or foods, causing vomiting, diarrhea, and abdominal pain.

Infectious gastroenteritis and food poisoning caused by noroviruses occur throughout the year but are particularly prevalent in winter.

**Norovirus prophylaxis**

A central heat of 85°C or more for more than 90 seconds is effective for foods that may be contaminated with norovirus.

Handwashing is the most effective way to reduce norovirus adhering to the fingers.

(Positive Frame 2) *Thorough preventive measures can help reduce norovirus infection among people.*

(Negative Frame 2) *Without thorough preventive measures, norovirus infection can spread rapidly among people.*

**Of note**

Since no antiviral medication is effective against the norovirus, symptomatic treatment is usually given. Severe dehydration may require treatment, such as administration of fluids in a hospital.

(Positive Frame 3) *Adequate hydration and nutrition can prevent dehydration and physical exhaustion.*

(Negative Frame 3) *Inadequate hydration and nutrition can lead to dehydration and physical exhaustion.*

**If you contract the norovirus**

Talk to your doctor or visit the nearest health center.

It is important to confirm the diagnosis and provide appropriate symptomatic treatment as soon as the outbreak occurs in nurseries, schools, facilities for the elderly, etc., and to investigate the route of infection and prevent the spread of infection.

(Positive Frame 4) *If you manage norovirus quickly, outbreaks can be prevented.*

(Negative Frame 4) *If you do not manage norovirus quickly, outbreaks will occur.*
